# Post-training Meditation Promotes Motor Memory Consolidation

**DOI:** 10.3389/fpsyg.2016.01698

**Published:** 2016-11-01

**Authors:** Maarten A. Immink

**Affiliations:** School of Health Sciences, Centre for Sleep Research and Cognitive Neuroscience Laboratory, University of South Australia, AdelaideSA, Australia

**Keywords:** Meditation, memory consolidation, motor learning, sequence learning, human performance, learning, memory

## Abstract

Following training, motor memory consolidation is thought to involve either memory stabilization or off-line learning processes. The extent to which memory stabilization or off-line learning relies on post-training wakeful periods or sleep is not clear and thus, novel research approaches are needed to further explore the conditions that promote motor memory consolidation. The present experiment represents the first empirical test of meditation as potential facilitator of motor memory consolidation. Twelve adult residents of a yoga center with a mean of 9 years meditation experience were trained on a sequence key pressing task. Three hours after training, the meditation group completed a 30 min session of yoga nidra meditation while a control group completed 30 min of light work duties. A wakeful period of 4.5 h followed meditation after which participants completed a test involving both trained and untrained sequences. Training performance did not significantly differ between groups. Comparison of group performance at test, revealed a performance benefit of post-training meditation but this was limited to trained sequences only. That the post-training meditation performance benefit was specific to trained sequences is consistent with the notion of meditation promoting motor memory consolidation as opposed to general motor task performance benefits from meditation. Further, post-training meditation appears to have promoted motor memory stabilization as opposed to off-line learning. These findings represent the first demonstration of meditation related motor memory consolidation and are consistent with a growing body of literature demonstrating the benefits of meditation for cognitive function, including memory.

## Introduction

Motor memory consolidation has been described as processes that provide for either motor memory stabilization or further off-line learning in the period that follows training (Walker et al., 2003a; Robertson et al., 2004a; Press et al., 2005). Memory stabilization related consolidation has been demonstrated by reduced susceptibility to interference from exposure to other tasks. (Brashers-Krug et al., 1996; Shadmehr and Brashers-Krug, 1997; Muellbacher et al., 2002; Walker et al., 2003a). Consolidation resulting in off-line learning has been demonstrated as improvements in performance gains following a period of time that does not involve training (Walker et al., 2002, 2003a,b). This off-line learning form of consolidation appears to be specific to tasks or effectors that have been trained since off-line learning does not appear to benefit transfer performance for new tasks or untrained effectors (Fischer et al., 2002; Korman et al., 2003).

Whether consolidation processes reduce susceptibility interference from competing memories or provide for off-line learning has been argued to depend on temporally dissociable stages of consolidation (Walker et al., 2003a,b; Walker and Stickgold, 2004). Training initiates learning resulting in large improvements in performance. However, following training, nascent motor memory is thought to be in a fragile state due to susceptibility for disruption, competition or interference (Brashers-Krug et al., 1996; Shadmehr and Brashers-Krug, 1997; Krakauer and Shadmehr, 2006). In the wakeful period that follows training, between 10 min to 6 h (Shadmehr and Brashers-Krug, 1997; Walker et al., 2003a), motor memory is thought to undergo a first stage of consolidation where it is stabilized against interfering or competing memories. Importantly, this first stage does not result in further performance improvements but supports maintenance of performance relative to end of training levels. The second stage of consolidation is thought to occur during the period of sleep (Karni et al., 1994; Stickgold et al., 2000; Fischer et al., 2002; Walker et al., 2002) or napping (Mednick et al., 2003; Nishida and Walker, 2007) that follows training and it is this sleep-dependent consolidation stage that enhances motor memory providing off-line gains in performance. Despite the support for the two stages of consolidation, there is some debate against this view (Peigneux et al., 2005; Brawn et al., 2010). Even if motor memory has undergone stages of consolidation that stabilize and enhance the memory, motor memory may once again be rendered fragile to interference by re-introduction of training, involving recall from long-term memory, and this process of re-training, memory instability and memory re-consolidation is thought to be important for the ongoing development and refinement of motor skills (Walker et al., 2003a; Monfils et al., 2009).

Further research is needed to provide a greater understanding of motor memory consolidation. For example, it is not yet clear how motor memory is stabilized following training and what factors mediate these stabilization processes (Korman et al., 2007). The introduction of memory interference following training has been the prevalent research paradigm used to address motor memory stabilization and clearly other research approaches are needed in order to gain a broader understanding of what memory stability entails. The common paradigm for investigating off-line learning has involved comparison of test performance to end of training performance with respect to whether a period of sleep or wakefulness occurred between training and test. Using this paradigm, studies have demonstrated that off-line motor performance gains rely on a period of sleep or more specifically, on certain stages of sleep (Walker et al., 2002, 2003a,b; Walker and Stickgold, 2004). However, the requirement of sleep for off-line gains has been questioned and the effects of specific sleep stages on consolidation has been suggested to be dependent on the type of motor task (Stickgold, 2005; Marshall and Born, 2007; Squire, 2009). The uncertainty in this literature includes demonstrations of off-line gains when only a wakeful period has followed training (Denny et al., 1955; Cohen et al., 2005; Brown and Robertson, 2007). Further, sleep has not always provided off-line gains (Brawn et al., 2010).

Another debate that has surrounded motor memory consolidation relates to whether or not the participant practiced with awareness of underlying motor task features or what task features are being learned. It has been proposed that when motor tasks are practiced under explicit conditions or with awareness of task features, motor memory consolidation requires a period of sleep (Robertson et al., 2004b). In contrast, practice under implicit conditions or with little or no awareness of task features, a wakeful period following practice is sufficient for consolidation (Robertson et al., 2004b, 2005; Press et al., 2005). However, not all findings align with this notion as consolidation of implicitly learned motor tasks has been demonstrated after sleep (Maquet et al., 2003; Peigneux et al., 2003) and off-line performance gains after a wakeful period have been demonstrated with explicit motor practice conditions (Spencer et al., 2006). More broadly, delineation of implicit versus explicit learning has not been entirely clear (Cleeremans et al., 1998; Frensch and Runger, 2003) leading some to argue that awareness is not the key distinguishing factor between these modes of motor learning (Whittlesea and Dorken, 1997) while others have argued for abandoning this delineation altogether (Willingham and Preuss, 1995; Cleeremans, 1997). Rather than being distinct processes, it might be that implicit and explicit learning processes interact or work in parallel during motor task acquisition. For example, Willingham and Goedert-Eschmann (1999) demonstrated that sequence learning under explicit or implicit instruction conditions resulted in equivalent learning outcomes. Thus, it is clear that novel research approaches are needed to further the understanding of what motor memory consolidation entails and requires with respect to memory stabilization and off-line learning.

Meditation might represent a novel approach to further our understanding of motor memory consolidation. Meditation has been defined as a complex set of cognitive processes (Newberg and Iversen, 2003; Cahn and Polich, 2006; Sperduti et al., 2012; Malinowski, 2013; Nash and Newberg, 2013) that are brought under voluntary control in a comfortable, relaxed but alert state (Craven, 1989; Walsh and Shapiro, 2006). The unique and complex cognitive processes and states associated with meditation highlight the importance of investigating meditation as a valuable opportunity to further understand of brain, cognition and consciousness (Cahn and Polich, 2006; Raffone and Srinivasan, 2010).

Meditation has been shown to influence or enhance cognitive function (Cahn and Polich, 2006; Tang et al., 2007; Zeidan et al., 2010; Lippelt et al., 2014; Colzato et al., 2015a,b, 2016). Specifically for memory, regular meditators outperform demographically matched adults on both short and long-term memory tasks (Lykins et al., 2012). In addition, brief periods of meditation practice have been shown to benefit performance on memory tasks (Mrazek et al., 2013; Xin et al., 2013; Quach et al., 2016). Demonstrations that meditation specifically enhances memory suggest that engaging in meditation following training might benefit memory processes including those associated with motor memory consolidation.

Further support for the potential of meditation to benefit motor memory consolidation is based on studies identifying neurophysiological processes associated with meditation that seem particularly relevant to memory consolidation. For example, neuroimaging work by Kjaer et al. (2002) has demonstrated increases in striatal dopamine, a neurotransmitter thought to be important for regulation of cognitive function (Nieoullon, 2002), working memory (Cools and D’Esposito, 2011) and memory consolidation (Karunakaran et al., 2016), following a single session of meditation. Findings from Kruis et al. (2016), which investigated changes in dopamine activity based spontaneous eye blink rate, suggest that meditation effects on dopamine might require long term practice with meditation techniques. A second point of support for the role of meditation in memory consolidation is based on research investigating the effects of meditation on cortical activity using electroencephalography (EEG) techniques. This work has demonstrated increases in theta band frequencies in anterior and frontal regions (Baijal and Srinivasan, 2010; Lomas et al., 2014, 2015). These findings are of particular interest with respect to reports of post-training gains in motor performance following a bout of theta-wave training using EEG neurofeedback (Reiner et al., 2014; Rozengurt et al., 2016). Finally, a basis for considering a role of meditation in memory consolidation lies in findings linking meditation to increased activity in the hippocampus (Lou et al., 1999; Lazar et al., 2000; Newberg and Iversen, 2003; Luders et al., 2009), a region important for motor sequence memory consolidation (Albouy et al., 2008, 2012, 2013), including during wakefulness (Karlsson and Frank, 2009).

There is empirical evidence to suggest that experiencing meditation and its associated cognitive processes and states following training can lend benefits for motor memory consolidation. The present experiment set out to test this proposition by having experienced meditators complete a single-session of meditation in the hours that followed a bout of motor sequence learning. Later on the same day, test performance on trained and untrained (novel) sequences was compared to a group of experienced meditators, who did not complete meditation after training. It was predicted that if meditation provides for consolidation in terms of motor memory stabilization, then post-training meditation would benefit trained sequence performance relative to the control group and trained sequence performance in the meditation group would be comparable between the end of training and test. On the other hand, if meditation engenders consolidation related to off-line learning, then performance would be improve between the end of training and test when compared for those who completed meditation after training. Finally, if consolidation associated with meditation is specific to trained tasks, then the retention or improvement of performance would only be observed with trained sequences and not novel sequences.

## Materials and Methods

### Participants

Twelve right-handed individuals (seven females; aged 35.6 ± 9.9 years) participated in the present experiment, which was conducted at a yoga center located in New South Wales, Australia, where the participants resided. Participants were experienced meditators with a mean of 9.0 years (±8.6, range 2–35) of self-reported regular meditation practice and a mean of 190 min (±92.9, range 90–420) of self-reported weekly meditation practice. All participants provided written informed consent prior to initiating their participation and the research protocol for this study was approved by the University of South Australia Human Research Ethics Committee.

### Apparatus and Stimuli

Stimuli for the motor sequence task were presented on a 48.3 cm display with 1024 × 768 pixel resolution and a refresh rate of 75 Hz. A PC with Intel^®^ Core^TM^ 2 Quad Q8300 CPU processor running at 2.53 GHz running E-Prime 2 (Psychological Software Tools Inc., Sharpsburg, PA, USA) on Windows 7 controlled stimulus presentation and recorded key press responses via a QWERTY keyboard. Participants sat with a viewing distance of 60 cm to the display but this was not strictly enforced. All stimuli were presented a black background field. At the start of each trial, an alerting stimulus based on a row of six dashes ( _ _ _ _ _ _ ) was presented in the center of the screen. The alerting stimulus represented the spatial position of the three left hand response keys (S, D, F, pressed by the ring, middle and index fingers of the left hand, respectively) and the three right hand response keys (J, K, L, pressed by the index, middle and ring fingers of the right hand, respectively) and also indicated the location where the response stimulus would be subsequently presented. Each dash was 2° visual angle in length, the left and right set of dashes were spaced 4° apart and dashes within each set was spaced 1° apart. Following presentation of the alerting stimulus, the response stimulus was then presented and this consisted of a set of five digits, each 2° visual angle in size and numbering between 1 and 5. These digits were each presented above a corresponding key position (e.g., 5 1 3 - 4 2), representing the order that each response key was to be pressed so that the key position with a “1” above meant that key was to be pressed first, the position with a “2” was the second key to be pressed and so on up to the fifth key of the sequence was. The key position with a “-” above indicated that key was not to be pressed in the sequence.

### Procedure

**FIGURE 1:**
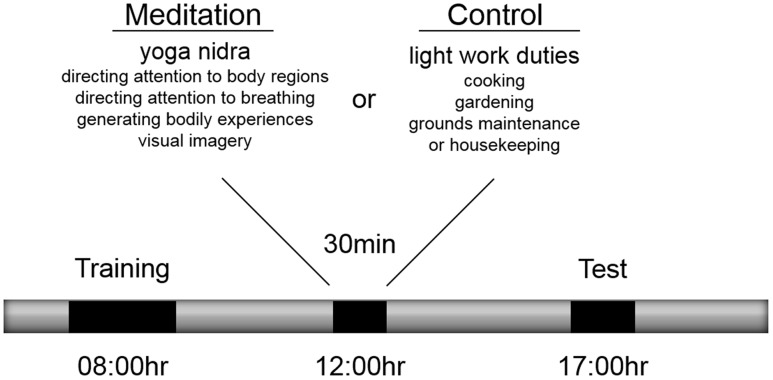
**Procedure for training, meditation and test phases**.

At 08:00 h (**Figure 1**) participants complete a training phase on the motor sequence task (Immink and Wright, 1998) in an administrative office of the yoga center that included a workstation with the apparatus and where the participant was alone with the experimenter. The commencement of the training phase was a mean of 3.1 (± 0.46) h after awakening from a mean of 7.0 (± 0.88) h of sleep. All participants had participated in a 90-min yoga class at 05:30 as was part of the yoga center’s daily routine. To start the training phase, participants received written instructions for the sequence production task. The instructions included information describing mapping of fingers with response keys and mapping of numeric digits with sequence ordering of response keys. In addition, the instructions indicated that 5-key sequences would be produced in each trial and that the aim of the task was to enter the sequence as accurately and as fast as possible. Following, presentation of instructions, participants completed eight familiarization trials using a practice sequence (S–L–D–K–F) to ensure participants understood the mapping between the stimuli and the sequence response. If by the end of the familiarization trials, participants could not accurately complete two trials, the familiarization trials were completed again. Next participants completed training on three unique sequences (D–L–F–K–S, K–D–L–F–J, J–S–K–D–L) over four blocks of 30 trials, where in each block, sequences were presented in a pseudo-random fashion based on randomizing the order every three trials without repetition of the same sequence on successive trials. At the start of the trial, participants were presented with a “Ready” message in the center of the screen for 2,000 ms. Next, the alerting stimulus was presented for a random delay period between 1,500 and 2,500 ms. Then, the response stimulus was presented and remained on the screen until the participant pressed the fifth key of the sequence. If one or more key presses in the sequence were incorrect, the participant received a response error message on the screen for 1,000 ms and the trial was repeated. Following accurate completion of the sequence, visual augmented feedback was presented for 1,500 ms on the monitor indicating that the response was accurate and also indicating their response time for the trial, which was based on the latency between presentation of the response stimulus and pressing the fifth key in seconds. While participant feedback involved response time, for the purpose of this experiment, sequence performance was recorded as reaction time (RT), the latency between response stimulus presentation and pressing the first key, and sequence entry time (SET), the latency between pressing the first key and the fifth key. Both RT and SET were recorded in milliseconds. A sixty-second rest interval was provided following blocks 1, 2, and 3. The time to complete the training phase was about 60 min.

Participants were randomly allocated to one of two experimental conditions that took place between 12:00 and 12:30 on the same day as the training and test phases. Participants allocated to the meditation condition participated in a 30-min yoga nidra meditation while participants allocated to the control condition participated in 30 min of light work duties (termed karma yoga) at various locations of the yoga center including the kitchen, gardening, grounds maintenance, and housekeeping. A historical account of yoga nidra meditation, existing descriptions of the technique and associated physiological correlates have been reviewed elsewhere by Parker et al. (2013). Based on the taxonomy proposed by Nash and Newberg (2013) and for the purpose of this study, this meditation was classified as being a cognitive-directed type of meditation because of its emphasis on purposefully attending to body sensations and generating body experiences and visual imagery. Its aim is described as inducing a state of deep relaxation while maintaining alertness. The 30-min technique includes eight stages: (1) preparation and internalizing attention, (2) mental repetition of a personal resolution statement or affirmation, (3) purposeful direction of attention to body regions, (4) awareness of sensations and experiences associated with breathing naturally, (5) imagining opposite body experiences (e.g., heavy vs. light, hot vs. cold), (6) visualization of natural scenes (e.g., a forest, waves on the beach), (7) mental repetition of a personal resolution statement or affirmation and (8) externalizing attention and closure as described by (Saraswati, 2001). Yoga nidra meditation was practiced while keeping the body still in a supine position with the eyes closed and verbal instructions were provided by an experienced instructor who also resided at the yoga center but who was not involved in the present experiment. Participants completed yoga nidra meditation in a group class format with other individuals who were also residents at the center. Participants in both conditions were informed that they would participate in one of two types of yoga activities between 12:00 and 12:30 but were not informed of what the alternative activity was. Except for their mid-day yoga activity, participants were instructed to not participate in any other type of yoga or meditation activity following the training phase.

At 17:00 h, participants completed a test phase involving performance of the three trained sequences and two untrained sequences (L-S-F-K-J, S-L-J-F-D) in a block of 20 trials with a pseudo-random order every five trials with a condition of no sequence repetition on successive trials. Reminder instructions about the task were presented in the test phase, and the trial procedure was the same as that described for the training phase with the exception that here, no response feedback was provided. Instead, participants were presented with an interval of 1,500 ms before the next trial. The test phase was about 20 min in duration.

## Results

### Group Differences in Participant Characteristics and Performance Error

To evaluate if random allocation of participants to meditation or control conditions resulted in group differences for participant characteristics, independent *t*-test analyses were conducted indicating no significant group differences for age (*p* = 0.13), years of self-reported meditation experience (*p* = 0.25), and weekly self-reported volume of meditation practice (*p* = 0.12). Chi-square analysis indicated no significant group differences in gender distribution (*p* = 0.56). In addition, group differences for the number of error trials that were re-run in training was tested using independent *t*-test analyses. In training, the number of error trials for the meditation group (*M* = 7.2, *SD* = 5.2) was not significantly different than the number of error trials for the control group (*M* = 6.2, *SD* = 5.6; *p* = 0.76). Group differences in error trials for trained (meditation, *M* = 1.8, *SD* = 1.8; control, *M* = 2.3, *SD* = 2.4) and untrained (meditation, *M* = 1.5, *SD* = 0.83; control, *M* = 1.8, *SD* = 3.0) sequences at test was analyzed using a 2 (Group: meditation, control) × 2 (Sequence: trained, untrained) analysis of variance (ANOVA) with repeated measures on the second factor. This analysis revealed no significant main effects of Group (*p* = 0.71) or Sequence (*p* = 0.43) and no significant Group × Sequence interaction (*p* = 0.89). Accurate trials where RT or SET performance was 3 standard deviations above the participant mean were classified as outlier data and these trials were removed from further analyses. In training, 1% of the trials were removed while at test 1.3% of trials were removed.

### Training Performance

**FIGURE 2:**
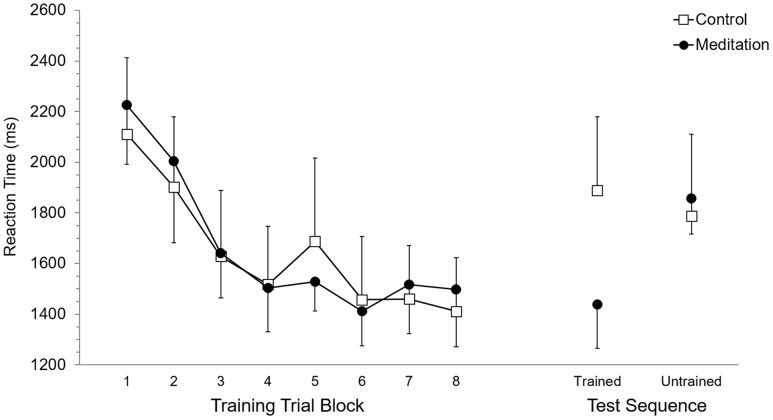
**Reaction time performance at training and test.** Error bars represent standard error of the mean.

**FIGURE 3:**
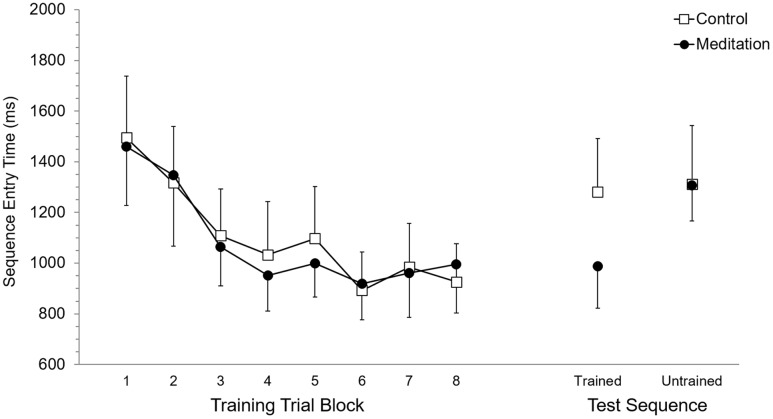
**Sequence entry time performance at training and test.** Error bars represent standard error of the mean.

Mean RT and SET for accurate trials was calculated for each participant according to eight trial blocks. As these eight trial blocks are based on dividing each of the four training blocks in half, they allowed evaluation of performance in the first half (or first 15 trials) versus the second half (trials 16–30) of each training block. Each of the eight trial blocks was based on 15 trials, or five trials of training on each of the three trained sequences. Participant mean RT and SET were separately submitted to 2 (Group: meditation, control) × 8 (Trial Block: 1–8) ANOVA with repeated measures on the second factor. For RT, the main effect of Group (*p* = 0.95) and the Group × Trial Block interaction (*p* = 0.89) were not significant while, the main effect of Trial Block was significant, *F*(7,70) = 13.7, *p* < 0.0001, $\eta_{p}^{2}$ = 0.58. *Post hoc* analysis using Duncan’s multiple range test identified the source of the main effect to be based on RT being significantly longer at Trial Block 1 and Trial Block 2 but RT was not significantly different between Trial Blocks 3 to 8. Analysis of SET revealed no significant main effect of Group (*p* = 0.94) and no significant Group × Trial Block interaction (*p* = 0.97). A significant main effect of Trial Block for SET, *F*(7,70) = 12.6, *p* < 0.0001, $\eta_{p}^{2}$ = 0.56, was based on significantly longer SET at Trial Blocks 1 and 2, which did not significantly differ, than subsequent Trial Blocks. RT and SET performance at training are presented in **Figures 2** and **3**, respectively.

### Test Performance

Mean RT and SET for accurate test trials was calculated for each participant for trained and untrained sequences. RT and SET were separately submitted to 2 (Group: meditation, control) × 2 (Sequence: trained, untrained) ANOVA with repeated measures on the second factor. Analysis of RT revealed no significant main effect of Group (*p* = 0.58) or Sequence (*p* = 0.11) while the Group × Sequence interaction was significant, *F*(1,10) = 8.35, *p* < 0.05, $\eta_{p}^{2}$ = 0.45. *Post hoc* analysis revealed that RT for the meditation group was significantly shorter for trained sequences (*M* = 1,437.9 ms, *SD* = 422.5) than for untrained sequences (*M* = 1,858.5 ms, *SD* = 346.8). Meditation group RT for untrained sequences was not significantly different than control group RT for trained (*M* = 1,888.0 ms, *SD* = 715.9) and untrained sequences (*M* = 1,786.7 ms, *SD* = 791.3), which also did not significantly differ. RT for trained sequences in the meditation group was significantly shorter than trained and untrained RT in the control group. For SET, the main effect of Group was not significant (*p* = 0.59) but the main effect of Sequence was significant, *F*(1,10) = 25.94, *p* < 0.001, $\eta_{p}^{2}$ = 0.72. This main effect was superseded by a significant Group × Sequence interaction, *F*(1,10) = 17,61, *p* < 0.01, $\eta_{p}^{2}$ = 0.64. For the meditation group, SET was significantly shorter with trained sequences (*M* = 988.0 ms, *SD* = 405.0) than untrained sequences (*M* = 1,307.0 ms, *SD* = 343.1) and was significantly shorter than SET for trained (*M* = 1,281,1 ms, *SD* = 515.4) and untrained (*M* = 1,311.9 ms, *SD* = 568.1) sequences in the control group. SET for untrained sequences in the meditation group and trained and untrained sequences in the control group did not significantly differ. RT and SET performance at test are presented in **Figures 2** and **3**, respectively.

### Trained Sequence Performance at Test Compared to End of Training

To compare test performance relative to end of training performance within each experimental group, the percentage change (Kuriyama et al., 2004) in RT and SET was separately calculated for each participant. The percentage change was calculated by subtracting trial block 8 from test performance, dividing by trial block 8 and then multiplying by 100, where positive percentage values reflect a proportional slowing in RT and SET at test and negative percentage values reflect performance gains (i.e., shorter sequence initiation and completion times) at test with these measures. Univariate analysis of the percentage change in RT revealed a significant Group effect, *F*(1,10) = 5.41, *p* < 0.05, $\eta_{p}^{2}$ = 0.35. The percentage change in RT for the meditation group (*M* = 0.2%, *SD* = 24.2) was significantly lower than the control group (*M* = 34.2%, *SD* = 26.3). Furthermore, the percentage change in RT for the meditation group was not significantly different than 0% (*p* = 0.99) while for the control group the percentage change in RT was significantly higher than 0% (*p* = 0.025). Univariate analysis of percentage change in SET between the meditation (*M* = 3.6%, *SD* = 30.5) and control (*M* = 37.7%, *SD* = 21.9) approached but did not reach significance (*p* = 0.052). The meditation group’s percentage change in SET did not significantly differ from 0% (*p* = 0.78) while percentage change in SET in the control group was significantly higher than 0% (*p* = 0.008).

## Discussion

The purpose of the present experiment was to investigate if meditation can promote motor memory consolidation processes following training. Three hours after completion of training on three key-pressing sequences, experienced meditators completed either a 30-min period of meditation or light work duties as the control condition. Then, 4.5 h after completion of meditation or control conditions, motor memory consolidation was tested with three previously trained and two untrained sequences.

Meditation does appear to promote motor memory consolidation since at test, trained sequence RT and SET was significantly shorter for the meditation group than the control group. RT reflects response planning processes and SET reflects response execution processes (Diedrichsen and Kornysheva, 2015) and in this case, both types of processes appear to have benefited from post-training meditation. More specifically, the observed benefits of post-training meditation for test performance can be explained by considering that meditation promoted memory consolidation to the extent that motor chunking was enhanced. Motor chunking, where successive movement elements are concatenated into a response unit (Verwey, 1996, 1999), is thought to be an important component of sequence learning and associated performance improvements (Boutin et al., 2010, 2014; Verwey and Abrahamse, 2012). The meditation group demonstrated significantly shorter RT and SET performance on trained sequences than on untrained sequences. In contrast, test performance in the control group did not significantly differ between trained and untrained sequences. This pattern of results suggests that the consolidation promoted by meditation was limited to previously trained sequences and did not afford transfer to the performance on untrained sequences, which is consistent with the notion that consolidation is specific to trained tasks and does not benefit transfer performance (Fischer et al., 2002; Korman et al., 2003). The absence of transfer effects in the meditation group is consistent with the interpretation that greater motor chunking was a product of the consolidation processes promoted by meditation. The performance benefit of motor chunking is sequence specific because concatenated response units are derived from the specific order of the movement elements that have been learned. The untrained sequences at test had different sequence structures to the trained sequences, which prevented utilization of trained sequence chunks with untrained sequences (Verwey et al., 2009).

That meditation group test performance on untrained sequences did not differ from control group test performance on trained and untrained sequences appears to rule out the explanation that meditation provided a general advantage for test performance, through greater alertness or processing capacity, for example. Had meditation provided general performance benefits then performance on both trained and untrained sequences would have favored the meditation group. Instead, the benefits of meditation for test performance are limited to trained sequences giving strength to the interpretation meditation promoted motor memory consolidation (Fischer et al., 2002; Korman et al., 2003). These results thus represent the first demonstration of motor memory consolidation following a single-session of meditation.

In the meditation group, performance on trained sequences at test is comparable to that present at the end of training. Thus, it is important to note that meditation did not promote consolidation in the sense of ‘off line’ performance gains (Walker et al., 2002, 2003a,b; Robertson et al., 2004a). That meditation did not provide ‘off line’ learning like sleep (Walker et al., 2002, 2003a,b) and wakeful periods (Denny et al., 1955; Cohen et al., 2005; Brown and Robertson, 2007) suggests that the form of consolidation observed at present following meditation is closer to the notion of stabilizing newly acquired information (McGaugh, 2000; Robertson et al., 2004a), which can occur independently from sleep (Donchin et al., 2002) in the first 6 h that follow training (Shadmehr and Brashers-Krug, 1997; Walker et al., 2003a). Because the test included both trained and untrained sequences, the potential existed for untrained sequences to interfere with the performance of trained sequences. This interference might explain why test performance in the control group did not differ between trained and untrained sequences and why trained sequence performance for the control group at test appears to revert back to levels observed at the start of training. The effects of interference at test might have been exacerbated for the control group by the fact that motor memory for trained sequences was rendered more fragile following memory recall activity necessary at test (Walker et al., 2003a; Monfils et al., 2009). Trained sequence test performance for the meditation group did not suffer from the same level of recall induced memory fragility or interference from untrained sequences because of the consolidation that the meditation afforded.

The present demonstration of motor memory consolidation effects following meditation are based on a small sample of experienced meditators, even though quite substantial effect sizes were observed in test effects (Levine and Hullett, 2002). The small sample reflected the limited availability of experienced meditators who resided at the yoga center at the time of this experiment. Inclusion of these residents was an advantage for the present experiment since for the most part, participants shared similar lifestyle behaviors such as regular practice of yoga and meditation and daily schedules in terms of waking, meal and sleep times. Accordingly and importantly, no differences in training performance were observed between groups. That consolidation effects following meditation were shown with experienced meditators brings in to question what extent of meditation experience or training might be required to derive these types of consolidation benefits. Future research should address this question as well as test the generalizability of these effects by including a larger and more representative sample.

Delineation of the mechanisms underlying meditation-based motor memory consolidation was beyond the scope of the present experiment but nonetheless the present findings pose important questions for future research. In the present experiment, it was assumed that those in the meditation group were able to reach high levels of engagement in the meditation technique given the high level of meditation experience in these participants. However, meditation engagement or depth of meditation experience was not objectively measured, which does somewhat limit interpretation of the influence of meditation on consolidation. In addition, it is not possible to rule out the possibility that participants might have slept during all or parts of the meditation, even as the explicit aim of the meditation technique is to remain awake and aware while following the instructions. Measurement of neural correlates of meditation, through EEG, for example, is thus needed in future work to characterize meditation as an agent for consolidation and to ensure consolidation effects are not attributable to those effects demonstrated with potentially similar agents such as napping (Mednick et al., 2003; Korman et al., 2007; Nishida and Walker, 2007).

## Conclusion

The present results provide the first demonstration of meditation-based promotion of motor memory consolidation in a wakeful period. Specifically, the introduction of meditation 3 h after training appears to have promoted motor memory stabilization as opposed to off-line learning. This stabilization was only evident in previously trained motor task variations suggesting that meditation-based promotion of motor memory consolidation does not support transfer performance. Research is needed to further investigate meditation promotion of motor memory consolidation with a larger sample size representing a range of meditation experience levels. Furthermore, the neural correlates of the meditation experienced after training need to be described in order to understand the underlying mechanisms by which meditation promotes motor memory consolidation.

## Author Contributions

MI was responsible for conceiving, developing, and conducting the experiment reported in this manuscript and MI drafted all sections of this manuscript.

## Conflict of Interest Statement

The author declares that the research was conducted in the absence of any commercial or financial relationships that could be construed as a potential conflict of interest.
